# First Experimental Quantitative Charge Density Studies of Advanced Intermediate of Vitamin D Analogues

**DOI:** 10.3390/molecules27061757

**Published:** 2022-03-08

**Authors:** Monika Wanat, Maura Malinska, Andrzej Kutner, Krzysztof Woźniak

**Affiliations:** 1 Biological and Chemical Research Centre, Department of Chemistry, University of Warsaw, 101 Żwirki i Wigury, 02-089 Warsaw, Poland; mwanat@uw.edu.pl (M.W.); mmalinska@chem.uw.edu.pl (M.M.); 2 College of Inter-Faculty Individual Studies in Mathematics and Natural Sciences (MISMaP), University of Warsaw, 2C Stefana Banacha, 02-097 Warsaw, Poland; 3 Department of Bioanalysis and Drug Analysis, Faculty of Pharmacy, Medical University of Warsaw, 1 Stefana Banacha, 02-097 Warsaw, Poland; andrzej.kutner@wum.edu.pl

**Keywords:** vitamin D, electron density, electrostatic interactions

## Abstract

Vitamins D are a group of fat-soluble secosteroids which play a regulatory role in the functioning of most cells. Rational design of new vitamin D analogs, of increased therapeutic potency and lowered calcemic side effects, requires high-resolution initial structures and a deep understanding of interactions with the molecular targets. In this paper, using quantum crystallography, we present the first determination of the experimental quantitative charge density of an advanced intermediate of vitamin D analogues as well as a reconstruction of the theoretical electron density of final vitamin D analogues. Application of these methods allows for topological and electrostatic interaction energy analysis. We showed that the A-ring chair conformation has a significant influence on the topological properties of vitamin D compounds. Moreover, the interactions between the CD-ring and side-chain additionally stabilize the crystal structure. These results are supported by our theoretical calculations and previous biological studies.

## 1. Introduction

**Figure 1:**
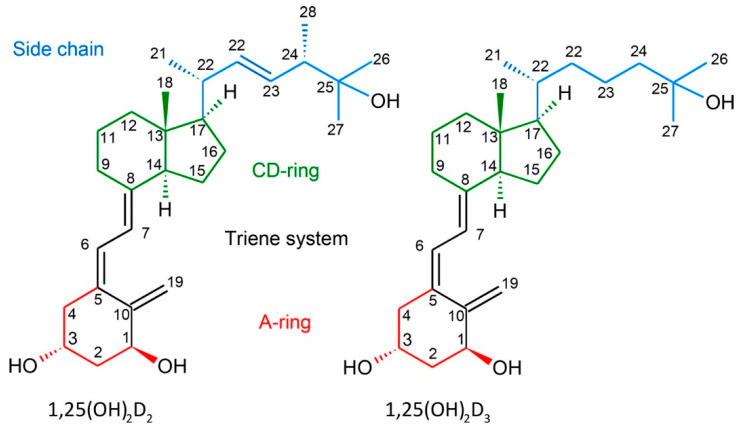
Numbering system and structural parts of 1,25(OH)_2_D_2_ and 1,25(OH)_2_D_3_. Reprinted with permission from Wanat et al. [10] Copyright © 2018 American Chemical Society.

**Figure 2:**
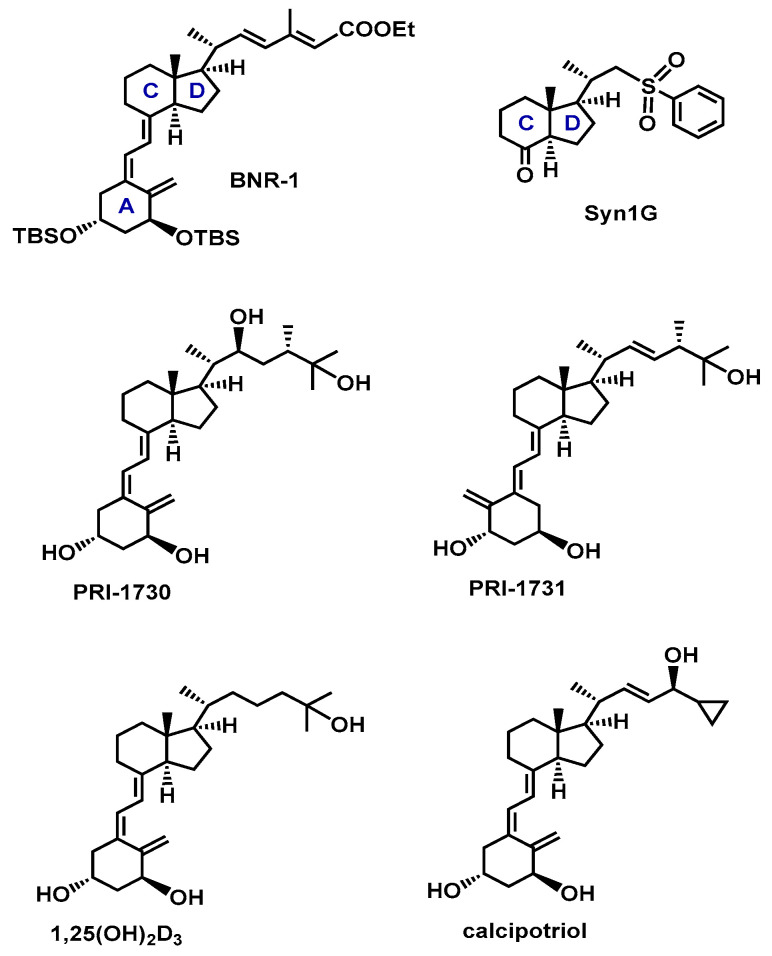
Structures of advanced (BNR-1) and basic (Syn-1G) intermediates of vitamin D as well as vitamin D_2_ analogues (PRI-1730, PRI-1731 and calcipotriol) and 1,25(OH)_2_D_3_ with the A and C, D rings labeled.

Vitamins D are a group of fat-soluble secosteroids where ergocalciferol (vitamin D_2_) and cholecalciferol (vitamin D_3_) are the two major forms activated by hydroxylations at the C-1 and C-25 carbon atoms (Figure 1). The most active form of vitamin D_3_, 1,25-dihydroxyvitamin D_3_ [1,25(OH)_2_D_3_], is a multifunctional hormone that regulates the homeostasis of calcium and phosphate. Moreover, the activity of vitamins D is mostly expressed through the binding to the vitamin D receptor (VDR). This protein belongs to the nuclear family of transcription factors and is heterodimerized by retinoid X receptor (RXR). This heterodimer enables binding to the hormone response elements which enables vitamin D influence on the genomic pathway. Very recently, we have demonstrated that it is the polymorphism of the VDR gene and not only the level of the total VDR that is responsible for the sensitivity of human leukemia and lymphoma cells to vitamin D analogues [1]. As a result of the wide presence of VDR in most systems, i.e., immune, muscle and circulatory, active vitamins D regulate the functions of most cells. For this reason, vitamin D analogues have found broad therapeutic applications, for instance, against secondary hyperparathyroidism (paricalcitol), and psoriasis (calcipotriol, Figure 2, and tacalcitol) [2,3,4,5,6]. Notably, in the synthetic structure of paricalcitol, 19-*nor* modification has been introduced (removal of 19-methylene), which reduces the hypercalcemic activity of vitamin D analogues [7]. Another example of 19-*nor* analogues are inecalcitol [8] and becocalcidiol (with additional 2-methylene group), which are currently in clinical trials [6] against prostate tumor and psoriasis, respectively. This shows the influence of the exocyclic methylene on the biological activity of vitamin D analogues and its importance in vitamin-D based drug design. Very recently, we first demonstrated that vitamin D analogues might be a new treatment for ovarian cancer [9].

In our previous X-ray studies of four new vitamin D analogues [10] we revealed that it was the hydrogen bond between 1- and 3-hydroxyls that led to the β-conformation of the A-ring. This conformation increases the biological affinity of these analogues for the VDR as compared with the analogues with the A-ring chair α-conformation. A-ring conformation is determined by mutual orientations of 1- and 3-hydroxyls. In the α-conformation the 1-hydroxyl adopts an axial orientation and the 3-hydroxyl an equatorial orientation, and the exocyclic methylene is below the plane of the A-ring. Opposite orientations of hydroxyls and exocyclic methylene occur in the A-ring β-conformation. The adoption of an α-conformation by vitamin D analogues is rare and related to the weaker biological activity and the higher energy in comparison to the β-conformation [11].

Moreover, we also evaluated the significant influence of the exocyclic 19-methylene on the properties of the vitamin D analogues and the nature of hydrogen bonds as this methylene in the natural position stabilizes the triene system [10]. Furthermore, our investigations of crystal structures of basic intermediates of vitamin D analogues revealed that the crystal structure of intermediates may allow for the prediction of structural properties of the crystal structure of the final analogue and its biological activity [12]. Both studies explain and illuminate the significant role of the position of exocyclic 19-methylene and its high impact on the biological activity of analogues.

Rational design of new vitamin D analogs, of increased therapeutic potency and lowered calcemic side effects, requires high-resolution initial structures and a deep understanding of interactions with the molecular targets. Therefore, in this work, we extend our studies on vitamin D analogues and present the first quantitative charge density analysis of an advanced intermediate of vitamin D (BNR-1, Figure 2) and a basic intermediate (Syn-1G, Figure 2). Experimental and theoretical charge density studies provide insights on intermolecular electrostatic interaction which may be essential in drug design as these forces influence the formation of the ligand–protein complexes. For instance, our charge density studies of vitamin D analogues and VDR complexes resulted in the design of four new agonists [13]. This shows that access to information about interactions may be further used in the design of new potential therapeutics. Therefore, one of the aims of this work is to extend the basic knowledge on the structural features of vitamin D analogues.

Up to now, the data collection of high-resolution XRD data of this class of compounds was not achieved. This is a result of poor quality and low scattering power of crystals of vitamin D analogues. Therefore, the experimental quantitative charge density studies for this group of compounds have not been performed, i.e., studies that require high-resolution data. This is the first work where high-resolution data of an advance intermediate of vitamin D is presented. Moreover, our research on quantum crystallography methods showed that quantum crystallography models may be used for the reconstruction of the electron density. The methodological correctness of this approach was proved by us in our previous studies [14,15]. Therefore, in this work we additionally present results of reconstruction of charge density of final crystal structures of vitamin D analogues (PRI-1730 and PRI-1731, Figure 2) which were obtained using the transferable aspherical atom model (TAAM). Crystal structures of BNR-1, PRI-1730 and PRI-1731 refined with the independent atom model (IAM) were published in our previous work [10]. Additionally, in this work, we present the analysis of topological properties and electrostatic interactions of 1,25(OH)_2_D_3_ [16] and calcipotriol [17] as examples of the natural metabolite and the drug substance, respectively.

This study is also an example that quantum crystallography methods may be used for the refinement of low-resolution data even when the quality of the data is not very high (in terms of measurement statistics and data resolution). When we performed the methodological work to prove this approach, we used the model compounds which did not exceed 25 atoms [14,15]. On the contrary, vitamin D analogues have more than 70 atoms. Some analogues described in the literature have even more than 100–120 atoms [18]. The number of atoms and their flexibility is a reason for difficulties with vitamin D crystallization. This is reflected in crystal and data quality. Therefore, this work is an example of the successful application of quantum crystallography methods for compounds with potential pharmaceutical importance.

## 2. Results and Discussion

### 2.1. X-ray Diffraction Studies

**Figure 3:**
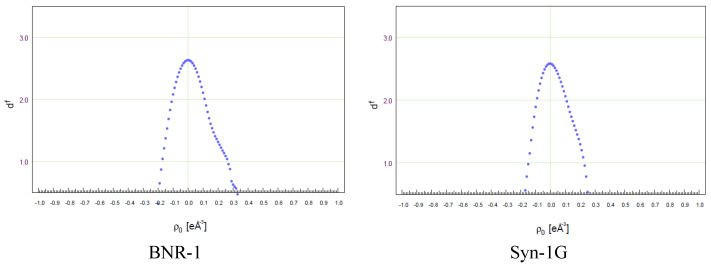

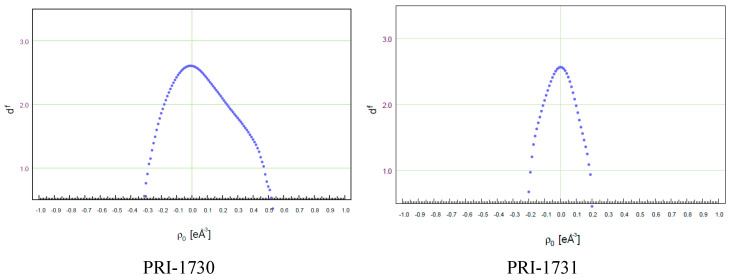
Fractal plots for BNR-1, Syn-1G, PRI-1730, PRI-1731.

Here, we report the crystal structures of two intermediate of vitamin D analogues (BNR-1 and Syn-1G) and two vitamin D analogues (PRI-1730 and PRI-1731) refined with quantum crystallography methods. The vitamin D intermediates (BNR-1 and Syn-1G) were refined with the multipole model, whereas the final vitamin D analogues (PRI-1730 and PRI-1731) with the TAAM model. The multipole model was applied for refinements against high-resolution data (d = 0.45 Å). On the contrary, the TAAM model was applied for refinements against low-resolution data (d = 0.81 Å). The collection of high-resolution data of vitamin D analogues (PRI-1730 and PRI-1731) was not achieved due to the low scattering properties of obtained crystals. The performed refinements for intermediates and vitamin D analogues were judged as reasonable. This is visible in the statistical parameters (Table 1) which are close to the statistical parameters after IAM refinement. Moreover, the fractal dimension plots (Figure 3) and residual density maps (Figure S2) show fitting experimental electron density to the model which confirms the final models.

We tried to perform the TAAM refinements of 1,25(OH)_2_D_3_ as examples of natural metabolite and calcipotriol which was approved as drug. However, structural data of these compounds are of poor quality and the application of TAAM refinement was not possible. Nonetheless, we transfered the pseudoatom parameters from the UBDB databank into these structures. Therefore, this allowed us insight into topological properties (see Section 2.3) and electrostatic interactions (see Section 2.4.3).

### 2.2. Non Covalent Interactions

**Figure 4:**
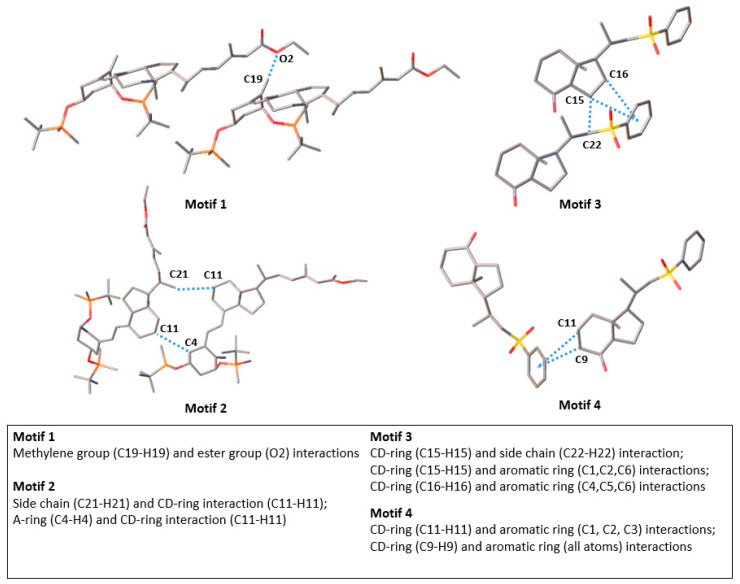
Selected interactions in BNR-1 (motifs 1 and 2) and Syn-1G (motifs 3 and 4). The most important carbon and oxygen atoms are captioned. The H-atoms were omitted for clarity; however, the numbering of H-atoms corresponds to the numbering of carbon atoms.

In the studies of vitamin D analogues, the hydrogen bonds formed by hydroxyls 1-OH, 3-OH and 25-OH significantly influence the intermolecular interaction in the crystal structure and the properties of vitamin D analogues. Hydroxyls are protected in BNR-1 and Syn-1G. Therefore, for these compounds, we analysed different non-covalent interactions and their role in stabilizing vitamin D derivatives’ crystal structures. We focused on the interaction of the exocyclic methylene group (motif 1) as it plays a major role in stabilizing the triene system (Figure 1) and has a significant influence on the biological properties as described in Section 1. Moreover, we included into considerations interactions between fragments of vitamin D (Figure 1). We chose interactions without protecting groups, as these groups are removed in the next steps of synthesis (motifs 2 and 3). Additionally, we analysed motifs 3 and 4 because the interactions between the CD-ring and the aromatic group are similar to those between the CD-ring and aromatic ring of Trp286, which belongs to the structure of VDR. Therefore, the most important interactions are presented in Figure 4:

- Motif 1: interaction between atoms C-19 and O-2 (BNR-1);
- Motif 2: interaction between atoms C-21 and C-11 as well as C-4 and C-11 (BNR-1);
- Motif 3: interaction between atoms C-15 and C-22, C-1, C-2, C-6 as well as C-16 and C-4, C-5, C-6 (Syn-1G);
- Motif 4: interaction between atoms C-11 and C-1, C-2, C-3 as well as C-9 and C-1, C-2, C-3, C-4, C-5, C-6 (Syn-1G).

We searched for selected motifs in previously analysed vitamin D analogues (PRI-1731, PRI-1730). Motif 3 involves the CD-ring interactions, and it was found in PRI-1731. However, the geometry of this interaction is different. The distance between the interacting atoms of motif 3 (H15…H22) is shorter for Syn-1G (2.348(6) Å) in comparison to PRI-1731 (2.868(9) Å). This may be a result of the stabilizing role of the C-H…π interactions with the aromatic ring in Syn-1G.

The CD-ring is crucial in the formation of the interaction with the conserved Trp286 and the correct placement of the agonist in the VDR binding pocket. Therefore, in the next step, we analysed the geometry of the interacting atoms of the CD-ring and Trp286. Then, we looked for similar interactions in the crystal lattice of the analysed compounds and found them in motifs 3 and 4. The interactions of the C-15 and C-16 atoms with the Trp286 were larger than 6 Å. These interactions occured in motif 3; however, these interactions did not exceed 3.75 Å. Therefore, the geometry of the interactions in motif 3 and the VDR complex are not related. Moreover, the distances between the interacting atoms C-9 and C-11 in the CD and Trip286 were in the range of 3.5–4.5 Å [19]. We found these interactions in motif 4 in the range 3.4–4.2 Å. Therefore, the interactions in motif 4 are related to the interactions between vitamin D and Trp286 in the complex with VDR contrary to the interactions in motif 3.

### 2.3. Charge Density Properties

**Figure 5:**
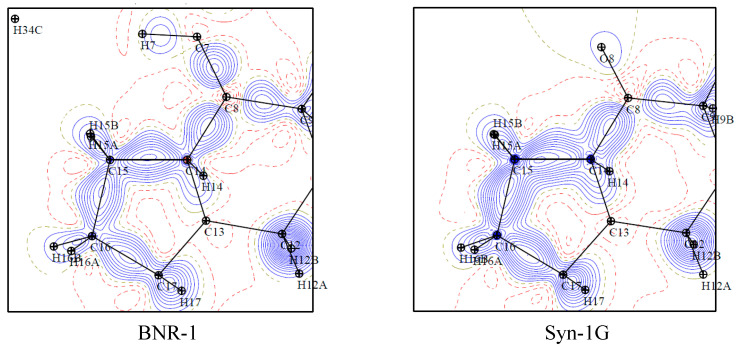

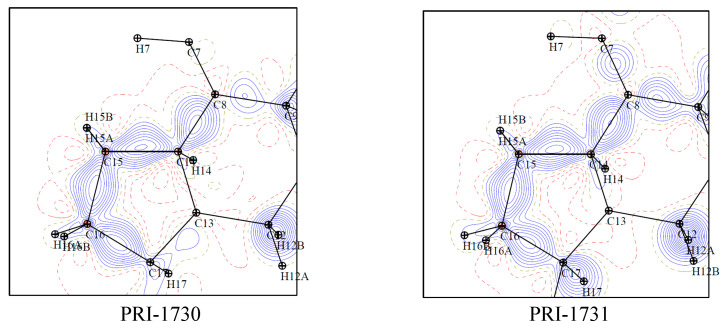
Deformation density maps for BNR-1, Syn-1G, PRI-1730 and PRI-1731. Maps are presented with contour levels with intervals of ±0.05 eÅ^−3^. Maps were prepared for planes determined by C-15 (center), C-14 (x axis) and C-16 (y axis) atoms.

We reconstructed the theoretical charge density distribution of vitamin D compounds (Figure 5 and Figure S1) with the aid of the UBDB. The PRI-1730 and PRI-1731 crystal structures were refined using TAAM, whereas the 1,25(OH)_2_D_3_ and calcipotriol crystal structures were analysed as they were after databank transfer. 1,25(OH)_2_D_3_ and calcipotriol were used as reference molecules. Details are described in Section 3.5. Therefore, based on the experimental (BNR-1, Syn-1G) or theoretical (PRI-1730, PRI-1731, 1,25(OH)_2_D_3_, calcipotriol) electron density, we calculated the critical points (see Section 3.6.3) and analysed selected bonds and interactions (Table 2, Tables S1 and S2).

#### 2.3.1. Experimental Electron Density

Firstly, the values of ρ(r) for bonds C1-O1 and C3-O3 are, respectively, the highest and the lowest for the BNR-1. This may be explained by the conformation of the A-ring, which is α for BNR-1 and β for other compounds, as well as by the influence of methylene, which increases the electron density of BCP at the bond which is closer to this group. This effect is significantly observed for the BNR-1 as the MM was applied. TAAM refinement of high-resolution data of BNR-1 resulted in the same values of electron density at the BCP of C1-O1 and C3-O3 bonds (ρ(r) = 1.68 eÅ^−3^). The influence of A-ring conformation is also reflected in the values of ρ(r) and ∇^2^ρ(r) of the bond with the methylene group, which is the highest and the lowest, respectively, for BNR-1.

#### 2.3.2. Theoretical Electron Density

Although the TAAM refinements use charge density parameters assigned based on the atom types from the databank, we can still observe some relation between the topological properties of corresponding bonds. Therefore, the opposite trend to the BNR-1 values of ρ(r) and ∇^2^ρ(r) was found for exocyclic methylene in PRI-1731, in which the methylene is in a modified position (attached to C-4 instead of C-10 carbon atom). The influence of the position of methylene on critical points of bonds with hydroxyls in the A-ring was not observed due to TAAM application (see Section 2.3.1). However, the differences were observed for intermolecular interactions with the methylene (Table S2). The values of ρ(r) for PRI-1730 and BNR-1 (compounds with a methylene group in the natural position) are in the range 0.04–0.06 eÅ^−3^, whereas for PRI-1731 these values are in the range 0.01–0.04 eÅ^−3^. Moreover, intermolecular interaction with exocyclic methylene has an influence on the topological properties of bonds with hydroxyls. For instance, the values of ρ(r) and ∇^2^ρ(r) for C1-O1 and C3-O3 for bonds of PRI-1730 were higher and lower, respectively, than for 1,25(OH)_2_D_3_. The opposite trend was visible for the C10-C19, C5-C6 and C7-C8 bonds, whereas the values for the C6-C7 bonds were almost identical. This may be explained by the difference in interactions with the triene system for synthetic PRI-1730 and natural 1,25(OH)_2_D_3_. Despite the presence of methylene in the same position, the triene system of PRI-1730 interacts with the CD-ring, whereas the triene system of 1,25(OH)_2_D_3_ interacts with the methylene. This suggests that not only the position of methylene but also modifications introduced to the side-chain significantly influence the interactions. This is also reflected in the topological properties.

### 2.4. Electrostatic Interaction Energy

#### 2.4.1. General Comparison

Firstly, we analysed intermolecular interaction in the most important motifs (chosen in Section 2.2). Motif 1 and motif 3 represent an interaction with exocyclic methylene and an interaction between the CD-ring and side-chain, respectively. (Figure 1) The interaction of these motifs has the lowest interaction energies among all analysed motif interactions in these two molecules based on the Gaussian computations (used as a reference, see Section 3.6.1) and energy framework calculations (Table 3, Figures S3–S8, see Section 3.6.2). This is not related to the distance between interacting atoms as the distance of interaction in motif 1 is equal to 2.62 Å, whereas distances in motif 2 are equal to 2.36 Å (H4B…H11B, motif 2) and 2.33 Å (H11B…H21A, motif 2) are close to the distance of H15…H22 interaction equal to 2.35 Å in motif 3.

#### 2.4.2. Energy Frameworks

According to the energy frameworks calculations (see Section 3.6.2), the presence of interactions between the CD-ring and side-chain led to lower interaction energy of motif HB1 (HB motifs available in Supplementary Materials, Figure S9) of 1,25(OH)_2_D_3_ (E_EF_ = −59.8 kJ/mol) in comparison with the energy of motif HB1 in PRI-1730 (E_Total_ = −55.1 kJ/mol). The energy of the HB1 motif was the lowest for the PRI-1731 (E = −62.6 kJ/mol). This may be a result of the presence of the additional interactions of the A-ring. These interactions stabilize the triene system instead of the methylene group in a natural position due to the absence of this group. Similarly, energy framework calculations showed a lower energy for the HB2 motif of PRI-1731 (E_Total_ = −67.5 kJ/mol) in comparison with 1,25(OH)_2_D_3_ (E_Total_ = −30.5 kJ/mol) as the HB2 motif of PRI-1731 is additionally stabilized by fragments interactions including CD-ring and side-chain interactions, whereas in the HB2 motif of 1,25(OH)_2_D_3_ it is only stabilized by the side-chain and A-ring interactions. Therefore, the interaction between fragments of vitamin D, i.e., CD-ring and side-chain, may stabilize the molecule, particularly when the triene system is not stabilized by the methylene in its natural position. This may be also reflected in the results of previous biological research. The binding affinity to the VDR for PRI-1731 is ten times higher than for PRI-1730, although the calculated electrostatic energy is lower for the PRI-1730 in comparison to PRI-1731. Similarly, the binding affinity to the VDR for PRI-1731 is five times lower in comparison to 1,25(OH)_2_D_3_ [11]. Unfortunately, the further comparison of these energies with the energies of these interactions in vitamin D analogues would not be reliable, as the presence of hydrogen bonds has a significant influence on interaction energies. For instance, the energy of interactions with the lowest energy (E_Total_ = −121.7 kJ/mol) was found in PRI-1730; however, these interactions include three different hydrogen bonds (HB3, HB7 and HB9). Although, the previous analysis showed that the hydrogen bond interactions have lower energy when they are additionally stabilized by the additional interactions between vitamin D fragments.

#### 2.4.3. Electrostatic Interaction Energy (E_es_ (EP/MM))

Analysis of electrostatic interactions (Table 4, see Section 3.6.3) in a cluster of molecules allows for the analysis of motifs with the interaction between the CD-ring and aromatic ring. The electrostatic interaction energy (E_es_ (EP/MM)) between a cluster of molecules and the aromatic ring is equal to E_es_ = −8 kcal/mol, which is two times larger than the interaction energy between the CD-ring and Trp286, which is equal to E_es_ = −4 kcal/mol [13]. Moreover, this analysis revealed the influence of introduced modification to the structure of vitamin D_3_ on the interaction energy of selected fragments. For instance, the formation of interactions between the CD-ring and side-chain decreased the total electrostatic energy of *frag3*, as the lowest energies for this fragment, i.e., E_es_ = −39 kcal/mol and E_es_ = −24 kcal/mol were obtained only for molecules PRI-1731 and Syn-1G, respectively, where interactions between the CD-ring and side-chain occur.

Furthermore, the methylene in a modified position (C-19 attached to C-4 instead of C-10, example of PRI-1731) as well as the presence of chair α-conformation (example of BNR-1) significantly increases the interaction energy of *frag1* and *frag2*. The presence of a double bond in the side-chain (example of PRI-1731) increases the interaction energy of *frag4*, as it was shown for the EB1 ligand with the complex with VDR. Interestingly, the presence of 22-OH and 28-Me (example of PRI-1730) in the side-chain only slightly increases the interaction energy of *frag4*. However, in this case the interaction energy of the whole molecule is significantly decreased. Moreover, the electrostatic interactions in a cluster of molecules are different than those in VDR. The highest differences between the interaction energy of 1,25(OH)_2_D_3_ and calcipotriol in cluster and VDR were observed for *frag1* and *frag3*, respectively.

## 3. Materials and Methods

### 3.1. Synthesis and Crystallization

Synthesis of and crystallization of the compounds were previously described: Syn-1-G, BNR-1 [10,20], PRI-1730, PRI-1731 [10,21].

### 3.2. Data Collection

Data collection of PRI-1730 and PRI-1731 was described in our previous paper [10]. Data of Syn-1G were collected using Bruker Ultra diffractometer (Bruker, Karlsruhe, Germany) equipped with Apex II detector and MoKα X-ray rotating anode. Data of BNR-1 were collected at the synchrotron facility SPring-8. Details of data collection are available in Table 5.

### 3.3. Structure Determination

The structure determination of PRI-1730 and PRI-1731 has been previously described [10]. Data reduction of Syn-1-G and BNR-1 were performed with the SAINT V7.60A and CrysAlis softwares (Rigaku Oxford Diffraction, Yarnton, UK), respectively. The structures of Syn-1-G and BNR-1 were solved with the direct methods using the Olex2 Software (OlexSys, Durham, UK) [22] and ShelXT program [23] using the IAM formalism. Refinements were performed with the aid of the ShelXL package [24] using least-squares minimization. The refinement was based on F^2^ for all reflections excluding negative intensities. Scattering factors were taken from the International Crystallographic Tables, Vol. C.28. All structures determined with IAM were used as a starting point for the further refinements, i.e., with the application of the TAAM and multipole model (MM).

### 3.4. Multipole Model

**Figure 6:**
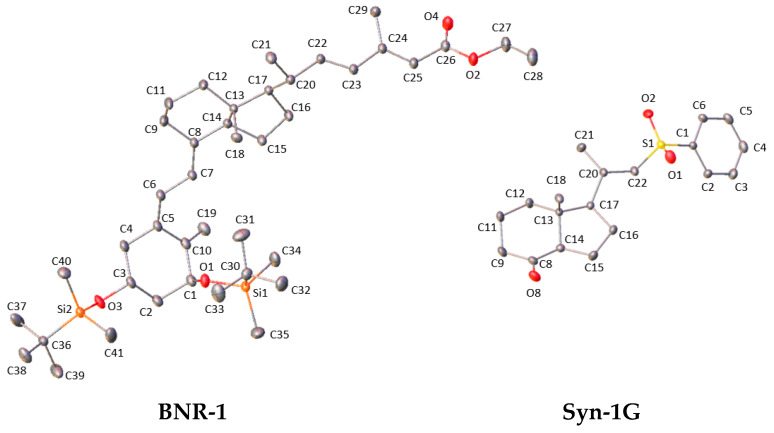
Displacement ellipsoids (50% probability level) of the molecular structure obtained with the MM refinements of BNR-1 and Syn-1G. H-atoms were omitted for clarity.

Starting from IAM, the multipole model was performed for Syn-1G and BNR-1 with the MoPro Software [25]. Firstly, for both structures the scale factor was adjusted and simultaneously refined with individually added position of all atoms and anisotropic ADPs of heavy atoms with isotropic ADPs of hydrogen atoms. Restrains applied for the isotropic H atom ADPs were equal to 1.2 with a standard deviation of 0.01, whereas restrains applied for X-H distances extended these values to the neutron ones [26] with standard deviation of 0.001. The first cycle of refinement was repeated for both structures; however, ADPs of hydrogen atoms were not refined and values of H-atoms APDs were constrained with constraints obtained from the SHADE3 server [27]. Restraints applied for X-H were the same as in the first cycle. In the third cycle the multipole parameters were simultaneously refined starting from monopoles, dipoles, quadrupoles and hexadecapoles. Hexadecapoles were refined only for the O and Si atoms. In the next step κ and both κ and κ’ were refined. Finally, anharmonicity was refined for selected atoms with the rest of the parameters fixed. For Syn-1G, the 3rd order of the anharmonic motion of S1 was refined. For BNR-1, the 3rd and 4th order of the anharmonic of Si-1, Si-2, C-37, C-38 and C-39 were refined. All described procedures were repeated for BNR-1 and two times for Syn-1G. Final structures are presented in Figure 6. (H-atoms ADPs are presented in Figure S1).

### 3.5. Transferable Aspherical Atom Model

**Figure 7:**
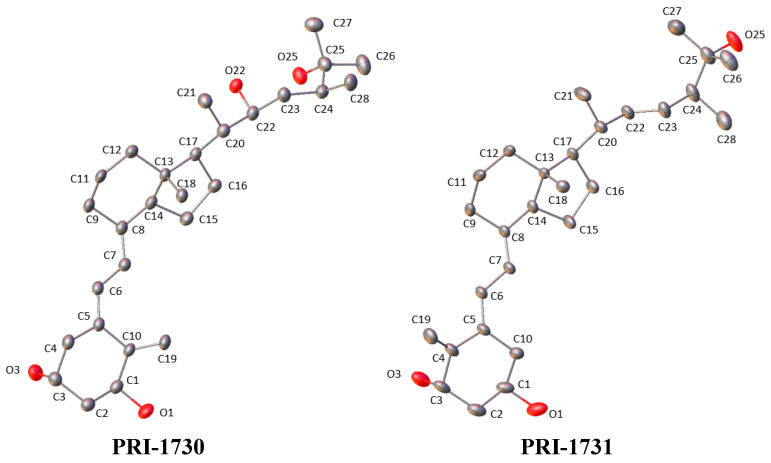
Displacement ellipsoids (50% probability level) of the molecular structure obtained with the TAAM refinements of PRI-1730 and PRI-1731. H-atoms were omitted for clarity.

Starting from IAM, TAAM refinements were performed for PRI-1730 and PRI-1731 [10]. Aspherical atom model parameters were transferred with the aid of the LSDB program [28] and the UBDB2016 databank [29]. Refinements were performed for the scale factor, position of all atoms, anisotropic ADPs of heavy atoms and isotropic ADPs of hydrogen atoms until convergence. Restraints were applied for the isotropic H atom ADPs (1.2 with standard deviation equal to 0.01) and X-H distances (lengths of bonds were extended to the neutron values [26] with standard deviation equal to 0.001). Obtained cif files were sent into the SHADE3 server [27] for the estimation of hydrogen atom ADPs. As a result, constraints were used in the next cycle of refinements as a replacement of previously used restraints of isotropic ADPs. When constraints were used, only the position of all atoms and ADPs of heavy atoms were refined. Final structures are presented in Figure 7 (H-atom ADPs are presented in Figure S5).

To extend analysis on structures of 1,25(OH)_2_D_3_ [16] and calcipotriol [17] we transferred the pseudoatom parameters from UBDB2016 [29] to the major component of molecular structure (the part with the highest occupancy factors for atoms) after elongation of the X-H bond lengths to averaged neutron values. Only this part of the crystal structure was included in further analysis. This procedure allows for reconstructing of the charge density distribution.

### 3.6. Theoretical Calculations

#### 3.6.1. Optimization and Interaction Energy

Calculations were performed with the Gaussian09 software (Gaussian, Inc., Wallingford, CT, USA) [30]. For all analysed molecules the geometry optimizations were performed with the DFT method, B3LYP functional and 6-311++g(2d,2p) basis set (B3LYP/6-311++g(2d,2p)). The interaction energy of the structural motifs was calculated with the DFT method, M06-2X functional and cc-pVDZ basis set. Functional M06-2X belongs to the Minnesota family and enables noncovalent interaction calculations, as the binding energy is the most accurate with this functional [31]. Standard counterpoise correction was applied to the basis set superposition error [32,33].

#### 3.6.2. Energy Frameworks

Calculations of energy frameworks were performed using Crystal Explorer 3.3 (DFT methods, B3LYP functional, 6-31G(d,p) basis set). Results for all frameworks were presented using a scale factor equal to 50 and a value of energy threshold equal to 5 kJ/mol. The view is along the X, Y and Z-axes. The energy frameworks for PRI-1730 and PRI-1731 were previously published [10].

#### 3.6.3. Electron Density Properties

Residual and deformation density maps, critical points and electrostatic interaction energy (E_es_) were based on charge density distribution based on a refined MM model (BNR-1 and Syn-1G), TAAM model (PRI-1730 and PRI-1731) or pseudoatom transfer (1,25(OH)_2_D_3_ and calcipotriol). Calculations were performed with the aid of the VMoPro module. Particularly, the E_es_ was calculated using the EP/MM method [34]. It combines a numerical evaluation of the exact Coulomb integral for short-range interatomic interactions (less than 4.5 Å) with a Buckingham-type multipole approximation for the long-range contacts. The calculations were performed for analysed fragments (*frag1*: A-ring; *frag2*: triene system; *frag3*: CD-ring; *frag4*: side-chain, Figure 1) and all atoms in a cluster around the selected fragments. The results obtained were compared to the electrostatic energy of vitamin D analogues in complexes with VDR, where charge density was also reconstructed based on the UBDB parameters [13].

## 4. Conclusions

In this work, we first presented multipole model refinements of intermediates of vitamin D analogues which allow for description of quantitative charge density. Moreover, we showed the application of TAAM for the reconstruction of theoretical charge density of vitamin D analogues, i.e., class of compounds with pharmaceutical importance for which SC-XRD studies are limited due to the poor crystal quality. Our results allow for the description of electrostatic energy, analysis of interactions, and topological properties of analysed compounds.

Chemical modification of the particular vitamin D fragments influences the topological properties of bonds in other fragments. For instance, the A-ring chair conformation has the strongest influence on the topological properties of the A-ring and triene system, showing again the importance of the A-ring conformation in vitamin D structure. Additionally, results of interaction energy calculations showed the stabilizing role of interactions between side-chain and CD-ring. These noncovalent interactions are particularly important for compounds without interactions between triene system and exocyclic methylene in the natural C-10 position.

## Figures and Tables

**Table 1:** Statistical parameters of MM refinements of BNR-1 and Syn-1G as well as TAAM refinements of PRI-1730 and PRI-1731.

Compound	BNR-1	Syn-1G	PRI-1730	PRI-1731
R[F^2^ > 2σ(F^2^)]	0.029	0.022	0.073	0.042
wR(F^2^)	0.058	0.040	0.139	0.082
number of reflections	35,507	20,082	5352	5323
number of fit parameters	634	286	427	456
Goodness of fit	1.07	1.05	1.52	1.52
Δρ_max_	0.34	0.25	0.53	0.19
Δρ_min_	−0.21	−0.17	−0.31	−0.21

**Table 2:** Selected topological properties of analysed bonds and intermolecular interactions for vitamin D analogues and intermediate.

Bond	Compound	R (Å)	ρ(r) (eÅ^−3^)	∇^2^ρ(r) (eÅ^−5^)
A-ring				
C1-O1	BNR-1	1.4328	1.82	−11.6
	1,25(OH)_2_D_3_	1.4360	1.71	−10.1
	calcipotriol	1.4514	1.66	−8.6
	PRI1730	1.4092	1.78	−12.3
	PRI1731	1.4265	1.73	−10.9
C3-O3	BNR-1	1.4331	1.69	−12.5
	1,25(OH)_2_D_3_	1.4309	1.73	−10.8
	calcipotriol	1.4363	1.71	−10.4
	PRI1730	1.4229	1.75	−11.4
	PRI1731	1.4224	1.74	−10.9
Methylene group				
C10-C19	BNR-1	1.3399	2.31	−21.8
	1,25(OH)_2_D_3_	1.3224	2.30	−22.0
	calcipotriol	1.3266	2.26	−21.1
	PRI1730	1.3312	2.27	−21.2
C4-C19	PRI1731	1.3416	2.24	−20.4
Triene system				
C5-C6	BNR-1	1.3527	2.31	−23.3
	1,25(OH)_2_D_3_	1.3139	2.30	−23.1
	calcipotriol	1.3431	2.21	−20.9
	PRI1730	1.3443	2.21	−20.6
	PRI1731	1.3453	2.20	−20.6
C6-C7	BNR-1	1.4577	1.92	−13.7
	1,25(OH)_2_D_3_	1.4531	1.93	−14.0
	calcipotriol	1.4551	1.92	−13.9
	PRI1730	1.4538	1.92	−14.0
	PRI1731	1.4554	1.92	−13.8
C7-C8	BNR-1	1.3504	2.32	−21.7
	1,25(OH)_2_D_3_	1.3295	2.27	−22.4
	calcipotriol	1.3381	2.24	−21.8
	PRI1730	1.3421	2.24	−21.6
	PRI1731	1.3482	2.22	−21.2

**Table 3:** Summary of interaction energies (kJ mol^−1^) of the most important motifs in BNR-1 and Syn-1G calculated with different methods. Methods are described in Section 3.6.1 (counterpoise), Section 3.6.2 (energy frameworks) and Section 3.6.3 (E_es_ (EP/MM)).

	BNR-1		Syn-1G	
	Motif 1	Motif 2	Motif 3	Motif 4
E (kJ/mol) (counterpoise)	−15.1	−7.6	−31.3	−14.8
E_Total_ (kJ/mol) (Energy frameworks)	−35.2	−25.0	−44.2	−20.5
E_Coulomb_ (kJ/mol) (Energy frameworks)	−8.0	−4.7	−21.0	−7.9
E_Dispersion_ (kJ/mol) (Energy frameworks)	−50.5	−37.9	−45.2	−22.3
E_es_ (EP/MM)	−51	−20	−26	−8

**Table 4:** E_es_ (kcal mol^−1^) calculated for the interaction between the analysed vitamin D analogue fragments (*frag1*–*frag4*) and the cluster of molecules based on the EP/MM method implemented in VMoPro. Details are in Section 3.6.3.

	*frag1*	*frag2*	*frag3*	*frag4*	*frag(1–4)*
PRI-1730	−53	−2	−8	−23	−98
PRI-1731	14	6	−39	−2	−35
BNR-1	0	0	−17	−5	−37
Syn-1G	n/a	n/a	−24	−46	−40
1,25(OH)_2_D_3_	−29	−1	7	−18	−46
1,25(OH)_2_D_3_ *	−62	−4	5	−20	−81
Calcipotriol	−33	−2	−7	−24	−66
Calcipotriol *	−42	−2	11	−19	−53

* E_es_ between vitamin D fragment and VDR residues [13].

**Table 5:** Experimental details for Syn-1G and BNR-1.

	Syn-1G	BNR-1
Empirical formula	C_5_H_26_O_2_S	C_41_H_70_O_4_Si_2_
Formula weight	334.46	683.15
Temperature/K	100.0(2)	80.0(2)
Crystal system	orthorhombic	orthorhombic
Space group	P2_1_2_1_2_1_	P2_1_2_1_2_1_
a/Å	7.8331(3)	9.6256(16)
b/Å	10.9261(4)	14.195(2)
c/Å	20.2743(9)	31.782(5)
α/°	90	90
β/°	90	90
γ/°	90	90
Volume/Å^3^	1735.18(12)	4342.5(12)
Z	4	4
ρ_calc_g/cm^3^	1.280	1.045
μ/mm^−1^	0.199	0.117
F(000)	720.0	1504.0
Crystal size/mm^3^	0.27 × 0.22 × 0.20	0.1 × 0.1 × 0.1
Radiation	Mo *K*α	Synchrotron (λ = 0.2482)
2Θ range for data collection/°	1.002 to 25.62	1.344 to 32.114
Index ranges	−17 ≤ h ≤ 15, −24 ≤ k ≤ 24, −45 ≤ l ≤ 43	−21 ≤ h ≤ 21, −31 ≤ k ≤ 31, −70 ≤ l ≤ 70
Reflections collected	150,739	354,339
Independent reflections	20,082 (R_int_ = 0.0271)	49,910 (R_int_ = 0.0651, R_sigma_ = 0.0346)
Data/restraints/parameters	20,082/26/286	49,910/0/438
Goodness-of-fit on F^2^	1.05	1.077
Final R indexes [I ≥ 2σ (I)]	R_1_ = 0.0215, wR_2_ = 0.0382	R_1_ = 0.0377, wR_2_ = 0.0959
Final R indexes (all data)	R_1_ = 0.0215, wR_2_ = 0.0396	R_1_ = 0.0564, wR_2_ = 0.1149
Largest diff. peak/hole/e Å^−3^	0.36/−0.19	0.44/−0.38
Flack parameter	0.004(7)	0.446(13)

## Data Availability

Crystallographic data of BNR-1 (MM; CCDC reference: 2156836), Syn-1G (MM; CCDC reference: 2156837), PRI-1730 (TAAM; CCDC reference: 2156839) and PRI-1731 (TAAM; CCDC reference: 2156838) are available.

## Supplementary Materials

The following supporting information can be downloaded at: https://www.mdpi.com/article/10.3390/molecules27061757/s1, Figure S1: Residual density maps for analysed compounds; Figure S2: Deformation density maps for analysed compounds.; Figure S3: Coulomb energy frameworks for BNR-1; Figure S4: Coulomb energy frameworks for Syn-1G; Figure S5: Dispersion energy frameworks for BNR-1; Figure S6: Dispersion energy frameworks for Syn-1G; Figure S7: Total energy frameworks for BNR-1; Figure S8: Total energy frameworks for Syn-1G; Figure S9: Selected motifs found in vitamin D analogues; Figure S10: Displacement Ellipsoids of analysed molecular structures; Table S1: Critical points for selected bonds of analysed compounds; Table S2: Critical points for selected intermolecular interactions of analysed compounds.

## Sample Availability

Samples of the compounds are available from the authors.
