# Supplementary material for: First Experimental Quantitative Charge Density Studies of Advanced Intermediate of Vitamin D Analogues
Source: Molecules. 2022 Mar 8;27(6):1757. doi: 10.3390/molecules27061757 (PMC8951618; doi:10.3390/molecules27061757)
Supplement: Supplementary file 1 [file molecules-27-01757-s001.zip › molecules-1607501-supplementary.pdf]

Supporting Information

First experimental quantitative charge density studies of advanced intermediate of vitamin D analogues

Monika Wanat[ab], Maura Malinska[a], Andrzej Kutner[c] and Krzysztof Wozniak[a*]

[a]Biological and Chemical Research Centre, Department of Chemistry, University of Warsaw, 101 Żwirki i Wigury, Warszawa, 02-089, Poland

[b]College of Inter-Faculty Individual Studies in Mathematics and Natural Sciences (MISMaP), University of Warsaw, 2C Stefana Banacha, Warszawa, 02-097, Poland

[c]Faculty of Pharmacy, Department of Bioanalysis and Drug Analysis, Medical University of Warsaw, 1 Stefana Banacha, Warszawa, 02-097, Poland

Correspondence email: kwozniak@chem.uw.edu.pl

List of content:

Table S1 Critical points for selected bonds of analysed compounds.

Table S2 Critical points for selected intermolecular interactions of analysed compounds.

Figure S1 Residual density maps for analysed compounds.

Figure S2 Deformation density maps for analysed compounds.

Figure S3-S8 Energy frameworks for BNR-1 and Syn-1G.

Figure S9 Selected motifs found in vitamin D analogues.

Figure S10 Displacement ellipsoids of analysed molecular structures

Table S1 Critical points for selected bonds of BNR-1, PRI-1730, PRI-1731 and 1,25$(OH)_2D_3$.

C1-O1	Gcp	Vcp	Gcp	Vcp	DIST12	DCP1	DCP2	DEN	LAPLACI	AN 3_HES	SIAN_EIG	EN-VALUES	ELLIPT	IC TYPE
BNR-1	0.24362	-0.60736	639.61	-1594.62	1.4328	0.8302	0.6027	1.82156	-11.58	-13.901	-12.571	14.893	0.1058	(3,-1)
HIJFAH	0.22677	-0.58022	595.37	-1523.36	1.4099	0.836	0.5762	1.7791	-12.212	-13.217	-12.784	13.789	0.0338	(3,-1)
PRI1730	0.22565	-0.57869	592.45	-1519.35	1.4092	0.836	0.5751	1.77688	-12.28	-13.113	-12.867	13.701	0.0191	(3,-1)
PRI1731	0.22236	-0.55791	583.82	-1464.8	1.4265	0.8352	0.5924	1.73272	-10.91	-12.791	-12.49	14.37	0.0241	(3,-1)
C3-O3	Gcp	Vcp	Gcp	Vcp	DIST12	DCP1	DCP2	DEN	LAPLACI	AN 3_HES	SIAN_EIG	EN-VALUES	ELLIPT	IC TYPE
BNR-1	0.20013	-0.52981	525.43	-1391.02	1.4331	0.8554	0.5778	1.69289	-12.489	-12.315	-12.29	12.116	0.002	(3,-1)
HIJFAH	0.22575	-0.58181	592.71	-1527.55	1.4092	0.5749	0.8363	1.78389	-12.561	-13.259	-12.87	13.568	0.0302	(3,-1)
PRI1730	0.22302	-0.56465	585.55	-1482.48	1.4229	0.8368	0.5883	1.74758	-11.433	-12.991	-12.601	14.16	0.0309	(3,-1)
PRI1731	0.22575	-0.5642	592.72	-1481.31	1.4224	0.5893	0.8343	1.74339	-10.863	-12.766	-12.451	14.354	0.0253	(3,-1)
CX-C19	Gcp	Vcp	Gcp	Vcp	DIST12	DCP1	DCP2	DEN	LAPLACI	AN 3_HES	SIAN_EIG	EN-VALUES	ELLIPT	IC TYPE
BNR-1	0.32956	-0.88515	865.26	-2323.97	1.3399	0.6808	0.6591	2.308	-21.788	-16.789	-14.222	9.222	0.1805	(3,-1)
HIJFAH	0.3167	-0.8474	831.5	-2224.84	1.3386	0.6494	0.6892	2.24724	-20.628	-16.728	-13.459	9.558	0.2429	(3,-1)
PRI1730	0.31959	-0.85963	839.07	-2256.97	1.3312	0.6444	0.6869	2.2683	-21.251	-16.92	-13.642	9.311	0.2403	(3,-1)
PRI1731	0.3151	-0.84176	827.29	-2210.03	1.3416	0.6511	0.6905	2.23776	-20.394	-16.646	-13.387	9.639	0.2434	(3,-1)
C5-C6	Gcp	Vcp	Gcp	Vcp	DIST12	DCP1	DCP2	DEN	LAPLACI	AN 3_HES	SIAN_EIG	EN-VALUES	ELLIPT	IC TYPE
BNR-1	0.31891	-0.87954	837.3	-2309.23	1.3527	0.6815	0.6713	2.30744	-23.3	-18.111	-14.596	9.407	0.2408	(3,-1)
HIJFAH	0.30853	-0.83921	810.04	-2203.33	1.3402	0.6675	0.6728	2.2392	-21.414	-16.615	-13.88	9.081	0.197	(3,-1)
PRI1730	0.3031	-0.81982	795.78	-2152.44	1.3443	0.6769	0.6683	2.20635	-20.593	-16.221	-13.643	9.272	0.189	(3,-1)
PRI1731	0.30239	-0.81829	793.94	-2148.43	1.3453	0.6697	0.6776	2.20401	-20.581	-16.249	-13.621	9.289	0.193	(3,-1)
C6-C7	Gcp	Vcp	Gcp	Vcp	DIST12	DCP1	DCP2	DEN	LAPLACI	AN 3_HES	SIAN_EIG	EN-VALUES	ELLIPT	IC TYPE
BNR-1	0.25979	-0.66127	682.07	-1736.17	1.4577	0.7281	0.7296	1.92284	-13.659	-14.146	-11.454	11.941	0.2351	(3,-1)
HIJFAH	0.25757	-0.65928	676.25	-1730.94	1.4531	0.7272	0.7262	1.92093	-13.895	-13.914	-11.322	11.341	0.229	(3,-1)
PRI1730	0.2579	-0.66088	677.13	-1735.15	1.4538	0.7268	0.7271	1.92404	-13.985	-13.94	-11.36	11.314	0.2271	(3,-1)
PRI1731	0.25717	-0.65795	675.21	-1727.46	1.4554	0.7279	0.7277	1.91847	-13.843	-13.874	-11.312	11.344	0.2265	(3,-1)
C7-C8	Gcp	Vcp	Gcp	Vcp	DIST12	DCP1	DCP2	DEN	LAPLACI	AN 3_HES	SIAN_EIG	EN-VALUES	ELLIPT	IC TYPE
BNR-1	0.33522	-0.8957	880.11	-2351.67	1.3504	0.6972	0.6534	2.32281	-21.715	-17.808	-13.898	9.991	0.2814	(3,-1)
HIJFAH	0.29899	-0.80473	784.99	-2112.82	1.3553	0.68	0.6764	2.18042	-19.931	-16.015	-13.533	9.618	0.1834	(3,-1)
PRI1730	0.30644	-0.83657	804.56	-2196.42	1.3421	0.6697	0.6728	2.23608	-21.563	-16.688	-13.969	9.095	0.1946	(3,-1)
PRI1731	0.30438	-0.82868	799.16	-2175.69	1.3482	0.6754	0.673	2.22257	-21.198	-16.589	-13.848	9.239	0.1979	(3,-1)

Table S2 Critical points for selected intermolecular interactions of of BNR-1, PRI-1730, PRI-1731 and Syn-1G.

		Gcp	Vcp	Gcp	Vcp	DIST12	DCP1	DCP2	DEN	LAPLACI	AN 3_HES	SIAN_EIG	EN-VALUES	ELLIPT
PRI1730	HB1 (O3…H1)	0.01942	-0.01706	51	-44.79	2.0002	1.2511	0.7492	0.14735	2.1	-0.733	-0.535	3.368	0.3711
	HB3 (O25…H3)	0.02816	-0.03102	73.92	-81.45	1.7965	1.1623	0.6343	0.24327	2.438	-1.415	-1.368	5.221	0.0342
	HB7 (O1…H22A)	0.03079	-0.03474	80.85	-91.22	1.7591	1.1445	0.6147	0.26343	2.587	-1.581	-1.568	5.736	0.0087
	Methylene (H9A…H19A)	0.00538	-0.00369	14.13	-9.68	2.2071	1.0959	1.1173	0.04441	0.682	-0.142	-0.117	0.941	0.2142
	H11B…H19A	0.00212	-0.00149	5.56	-3.92	2.4918	1.2446	1.2602	0.02705	0.264	-0.097	-0.073	0.434	0.3196
	C19…H21C	0.00115	-0.00079	3.02	-2.06	3.4302	1.977	1.4663	0.0175	0.146	-0.033	-0.021	0.2	0.586
	H16B…H19B	0.00422	-0.00335	11.08	-8.79	2.1793	1.0951	1.0911	0.05065	0.491	-0.191	-0.168	0.85	0.1374
PRI1731	HB1 (H1…O3)	0.02053	-0.01964	53.91	-51.56	1.9472	0.7156	1.233	0.17053	2.065	-0.891	-0.765	3.721	0.1638
	HB2 (H25…O1)	0.0287	-0.03285	75.36	-86.26	1.7733	0.6227	1.1506	0.25647	2.367	-1.53	-1.523	5.419	0.0046
	HB3 (O25…H3A)	0.02934	-0.03364	77.03	-88.33	1.769	1.1509	0.6182	0.26036	2.413	-1.571	-1.556	5.54	0.0092
	Methylene (H19B…H18C)	0.00306	-0.0021	8.03	-5.51	2.4751	1.2132	1.2748	0.03173	0.387	-0.101	-0.07	0.559	0.4409
	H19B…H12B	0.00394	-0.00273	10.33	-7.16	2.3199	1.2176	1.1507	0.0378	0.496	-0.138	-0.078	0.712	0.774
	H19A…H23	0.00136	-0.00082	3.58	-2.15	2.8001	1.4273	1.411	0.01364	0.184	-0.041	-0.024	0.249	0.6825
	H19B…H20	0.00348	-0.00243	9.14	-6.37	2.3734	1.2174	1.1656	0.03547	0.437	-0.131	-0.097	0.666	0.3456
	H15A…H22	0.00101	-0.00062	2.65	-1.62	2.8689	1.4462	1.4341	0.01192	0.135	-0.033	-0.027	0.195	0.1869
BNR-1														
dimer 1	Methylene (H19A…H33C)	0.00182	-0.00232	4.79	-6.1	2.6088	1.3271	1.2923	0.05478	0.128	-0.202	-0.199	0.529	0.0132
	H19B…O2	0.00451	-0.00347	11.85	-9.11	2.6244	1.0945	1.5328	0.04997	0.536	-0.173	-0.146	0.855	0.1871
dimer 2	C21…C11	0.00398	-0.0027	10.45	-7.08	2.3275	1.221	1.1159	0.03612	0.508	-0.136	-0.08	0.724	0.6959
	C4…C11	0.00351	-0.00213	9.22	-5.59	2.3594	1.1673	1.2072	0.02459	0.472	-0.103	-0.031	0.606	2.3629
Syn-1G														
dimer 3	C15…C22 (H22A…H15B)	0.00236	-0.00146	6.2	-3.83	2.3483	1.1831	1.1656	0.02063	0.315	-0.089	-0.055	0.459	0.6204

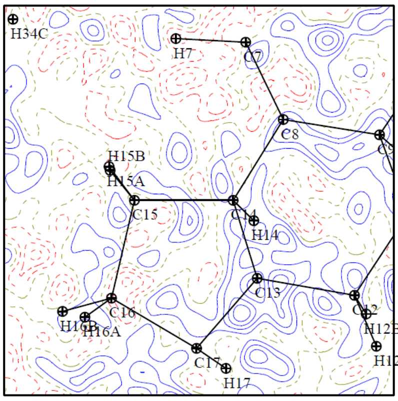

C15, C14, C16

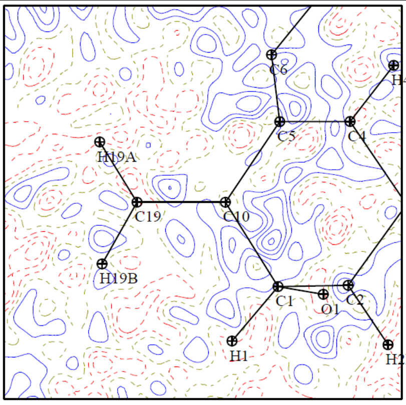

C19, C10, H19B

BNR-1

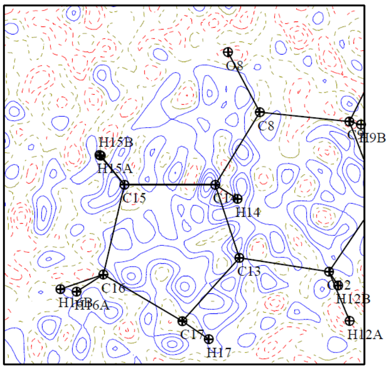

C15, C14, C16

Syn1G

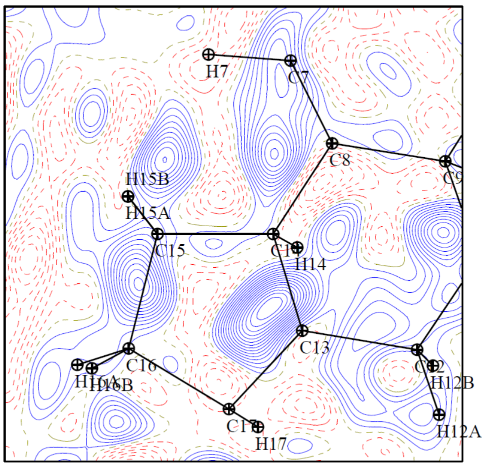

C15, C14, C16

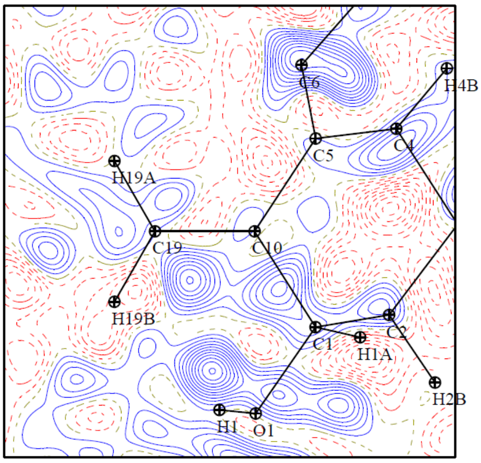

C19, C10, H19B

PRI-1730

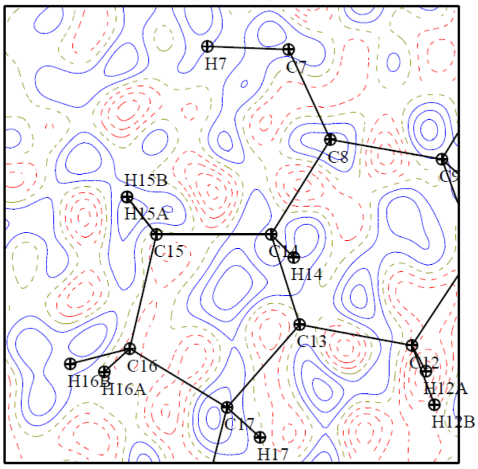

C15, C14, C16

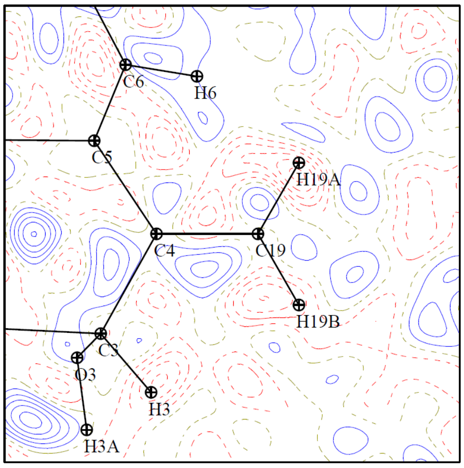

C19, C4, H19B

PRI-1731

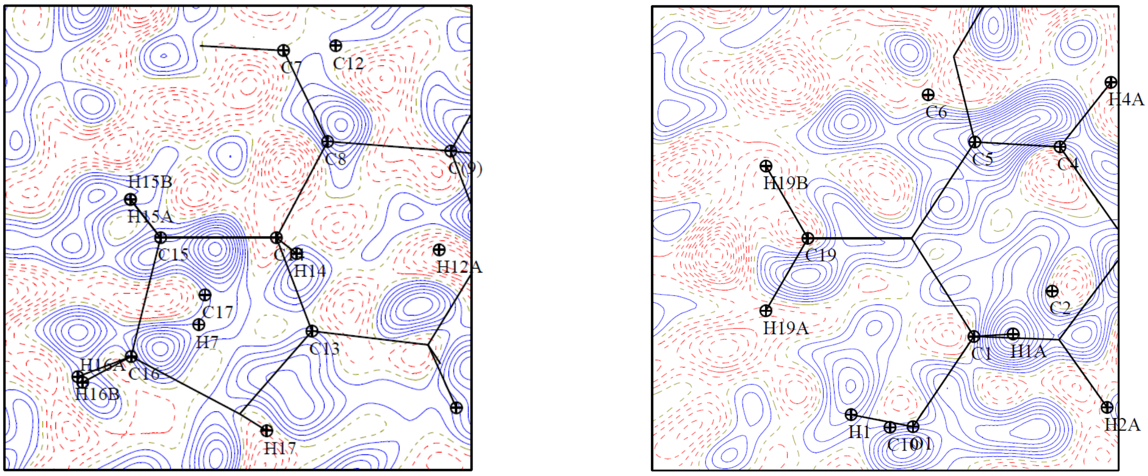

C15, C14, C16

C19, C10, H19B

1,25(OH)$_2$D$_3$

Figure S1 Residual density maps for BNR1, Syn1G, PRI-1730 and PRI-1731. Maps are presented with contour levels with intervals of ± 0.05eÅ^{-3}. Blue lines represent positive values and red lines negative values. Maps were prepared for planes determined by C15 (centre), C14 (x axis) and C16 (y axis) atoms or, excluding Syn1G, C19 (centre) C10 (x axis for BNR-1 and PRI-1730), C4 (x axis for PRI-1731) and H19B (y axis) atoms. Details are given below the maps.

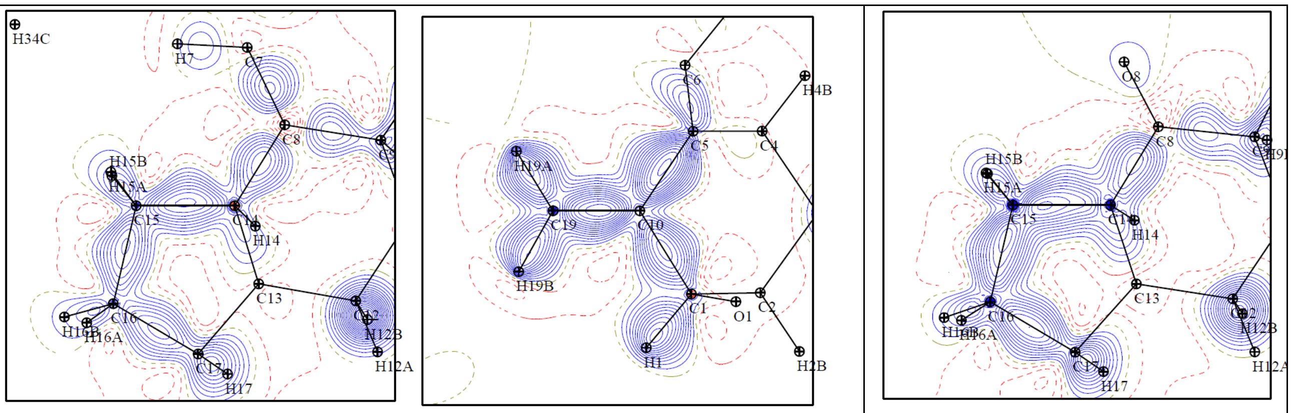

C15, C14, C16

C19, C10, H19B

BNR-1

C15, C14, C16

Syn1G

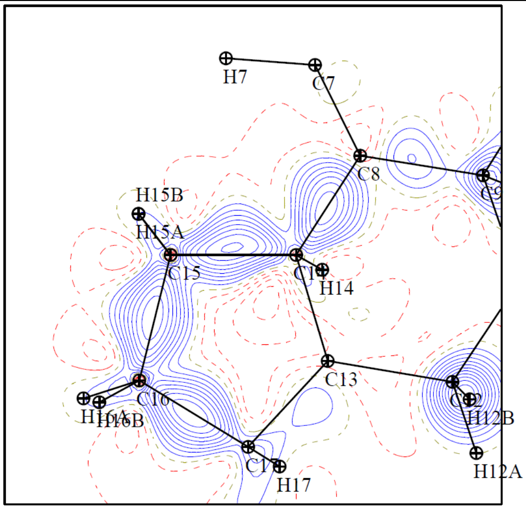

C15, C14, C16

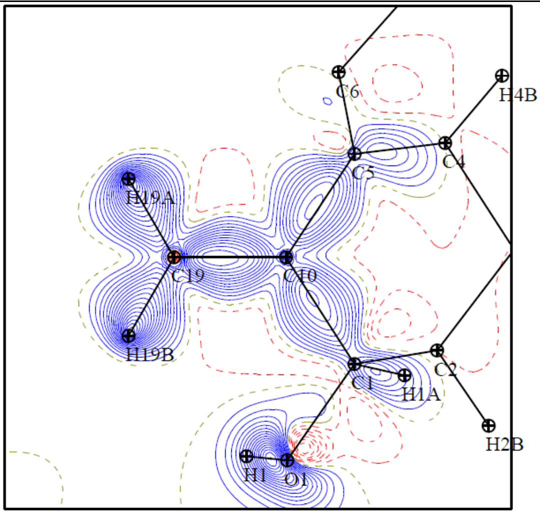

C19, C10, H19B

PRI-1730

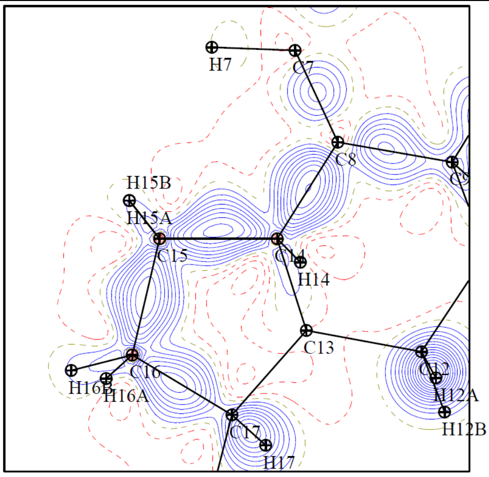

C15, C14, C16

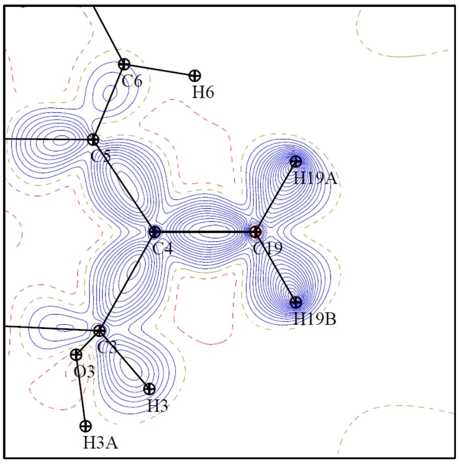

C19, C4, H19B

PRI-1731

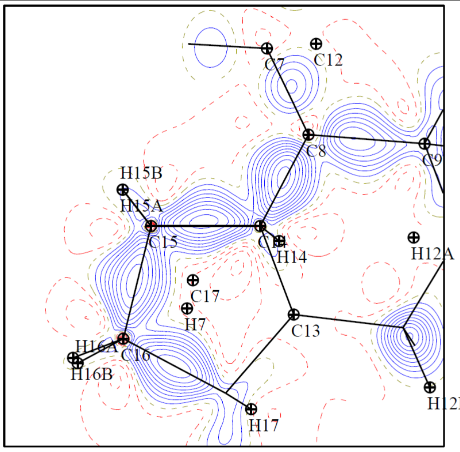

C15, C14, C16

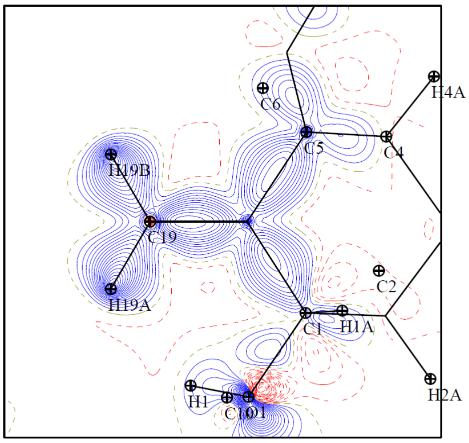

C19, C10, H19B

$1,25(OH)_2D_3$

Figure S2 Deformation density maps for BNR1, Syn1G, PRI-1730 and PRI-1731. Maps are presented with contour levels with intervals of ± 0.05eÅ^{-3}. Blue lines represent positive values and red lines negative values. Maps were prepared for planes determined by C15 (centre), C14 (x axis) and C16 (y axis) atoms or, excluding Syn1G, C19 (centre) C10 (x axis for BNR-1 and PRI-1730), C4 (x axis for PRI-1731) and H19B (y axis) atoms. Details are given below the maps.

Energy frameworks:

Calculations of energy frameworks were performed using Crystal Explorer 3.3 (DFT methods, B3LYP functional, 6-31G(d,p) basis set). Results for all frameworks were presented using scale factor equal 50 and value of energy threshold equal 5 kJ/mol. View along X,Y and Z axis. Energy frameworks for PRI-1730 and PRI-1731 were previously published (Wanat *et al.*, 2018).

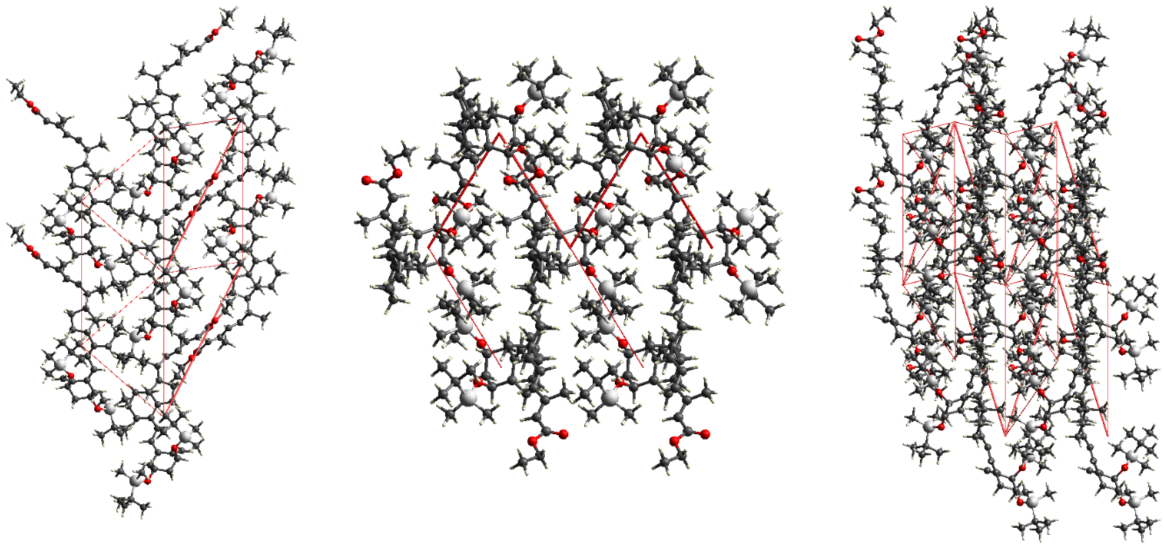

Figure S3 Coulomb energy frameworks for BNR-1

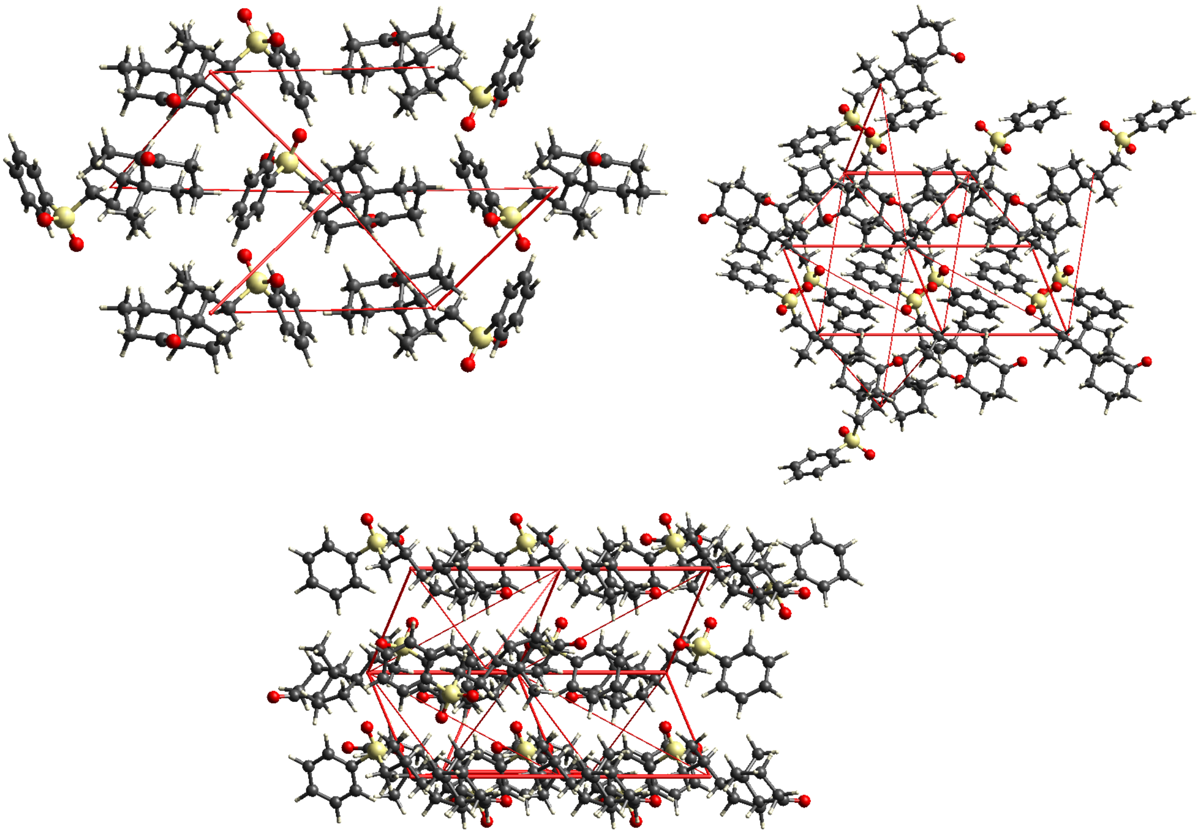

Figure S4 Coulomb energy frameworks for Syn1G.

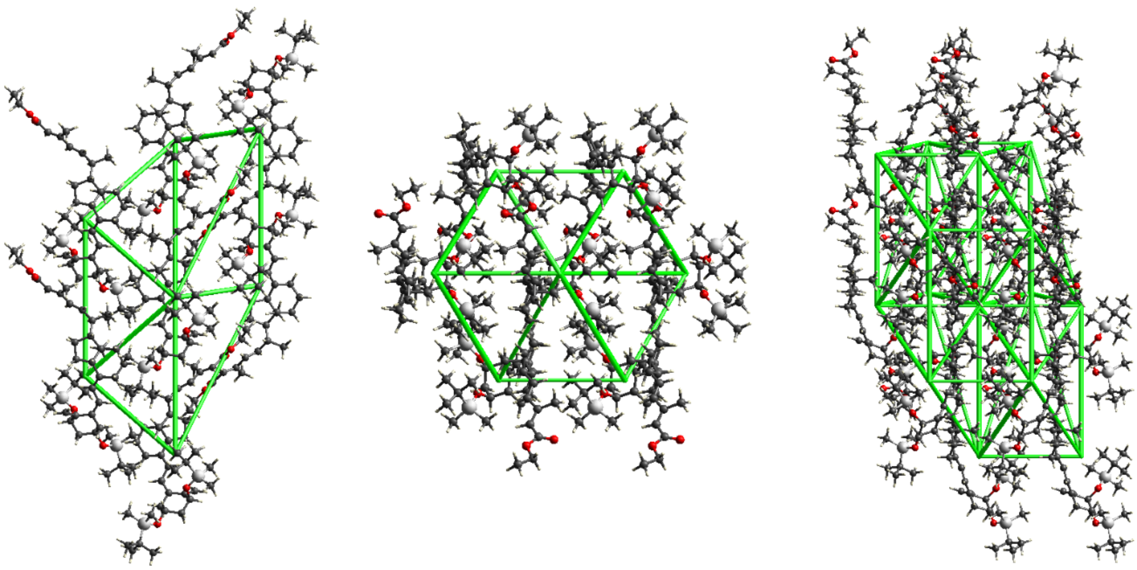

Figure S5 Dispersion energy frameworks for BNR-1

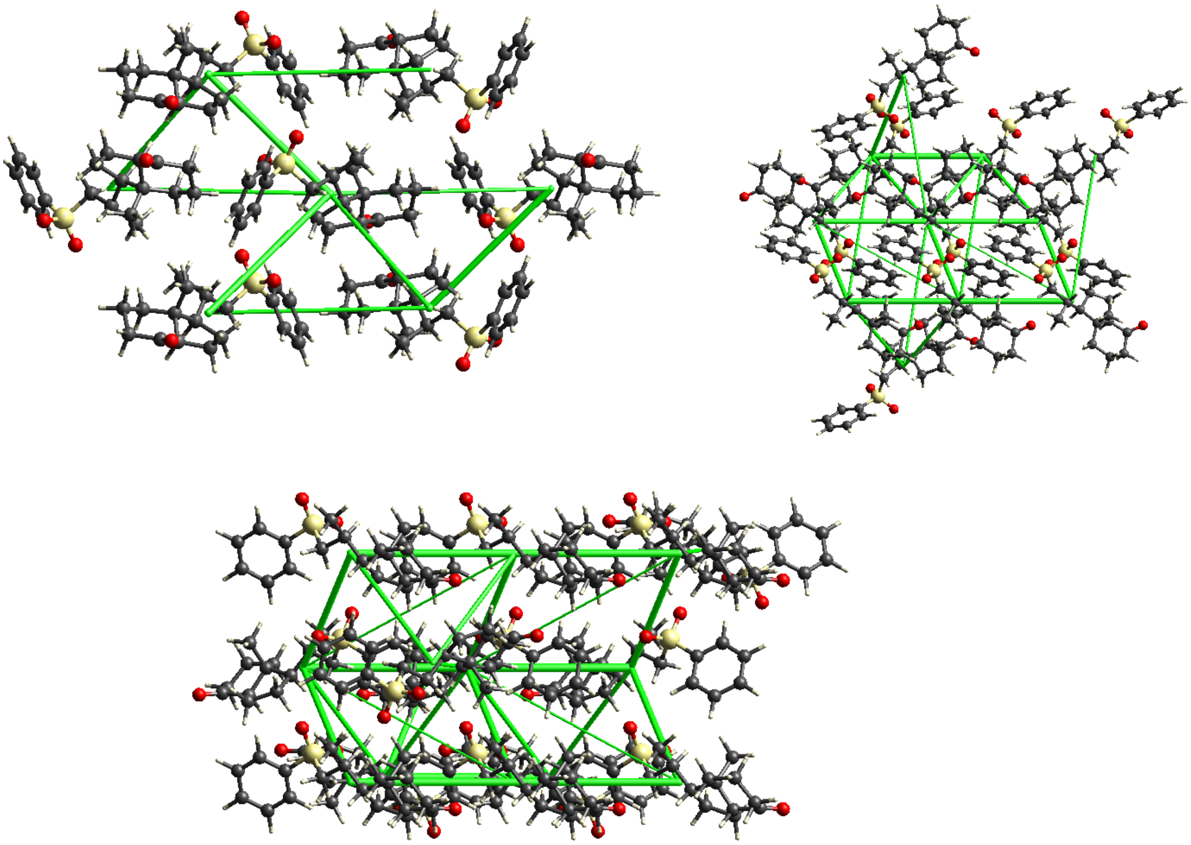

Figure S6 Dispersion energy frameworks for Syn1G.

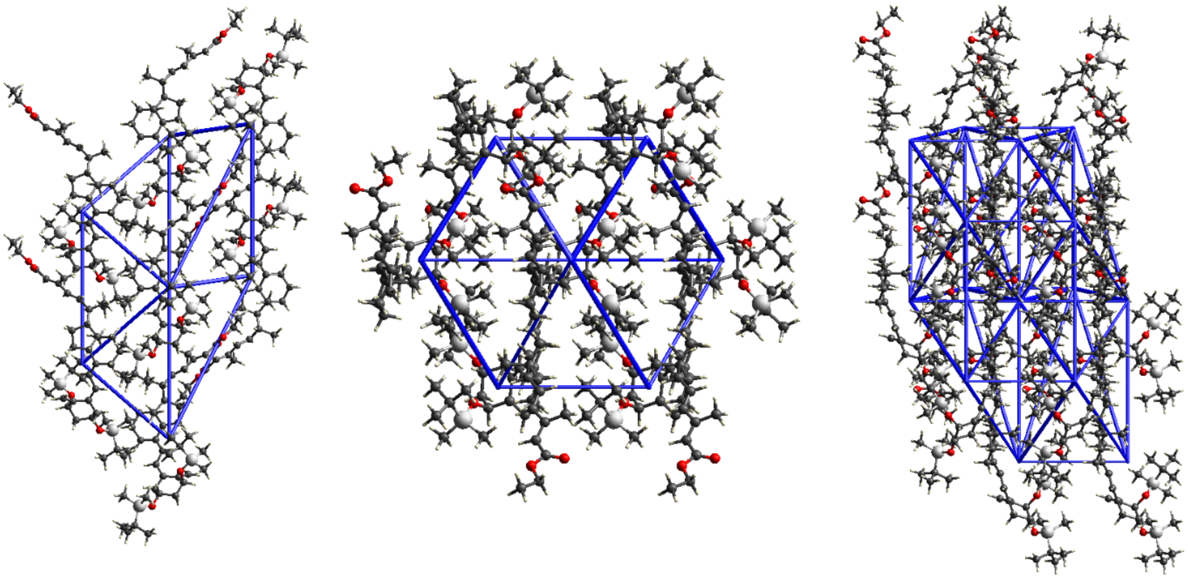

Figure S7 Total energy frameworks for BNR-1

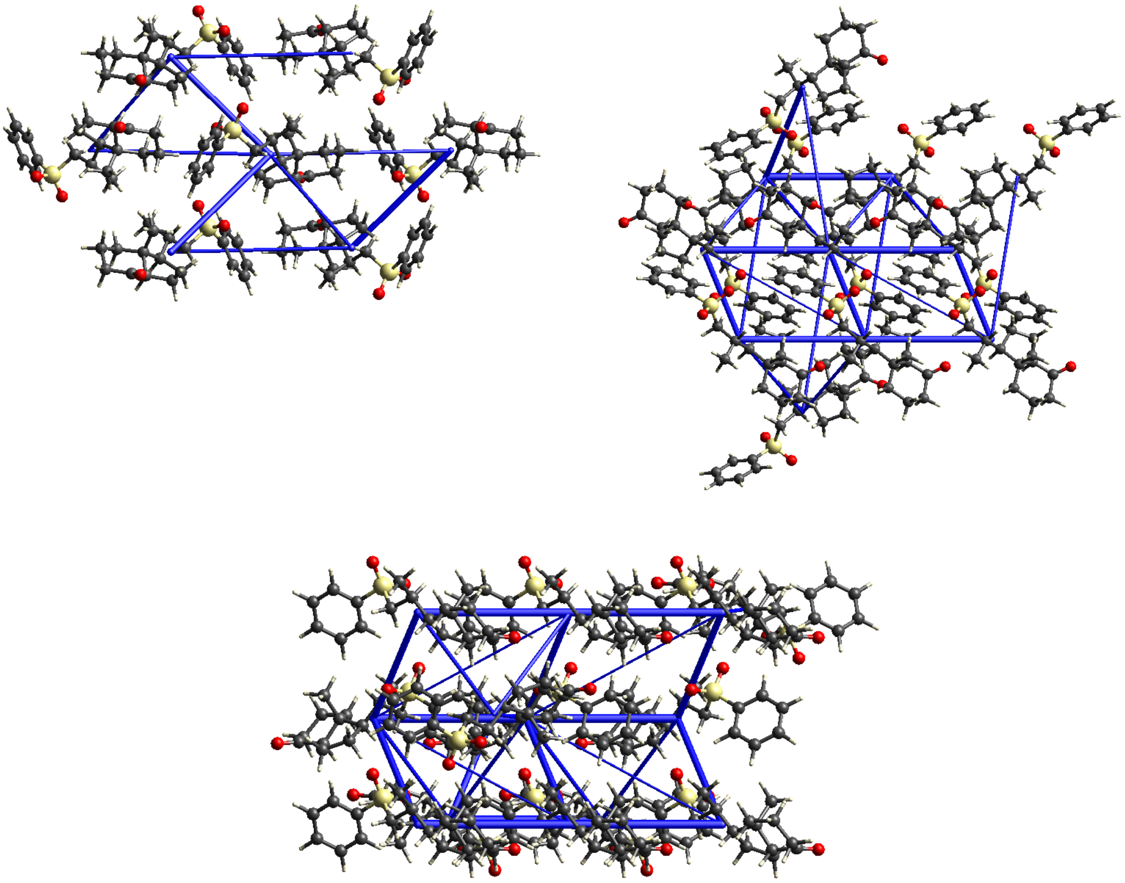

Figure S8 Total energy frameworks for Syn1G.

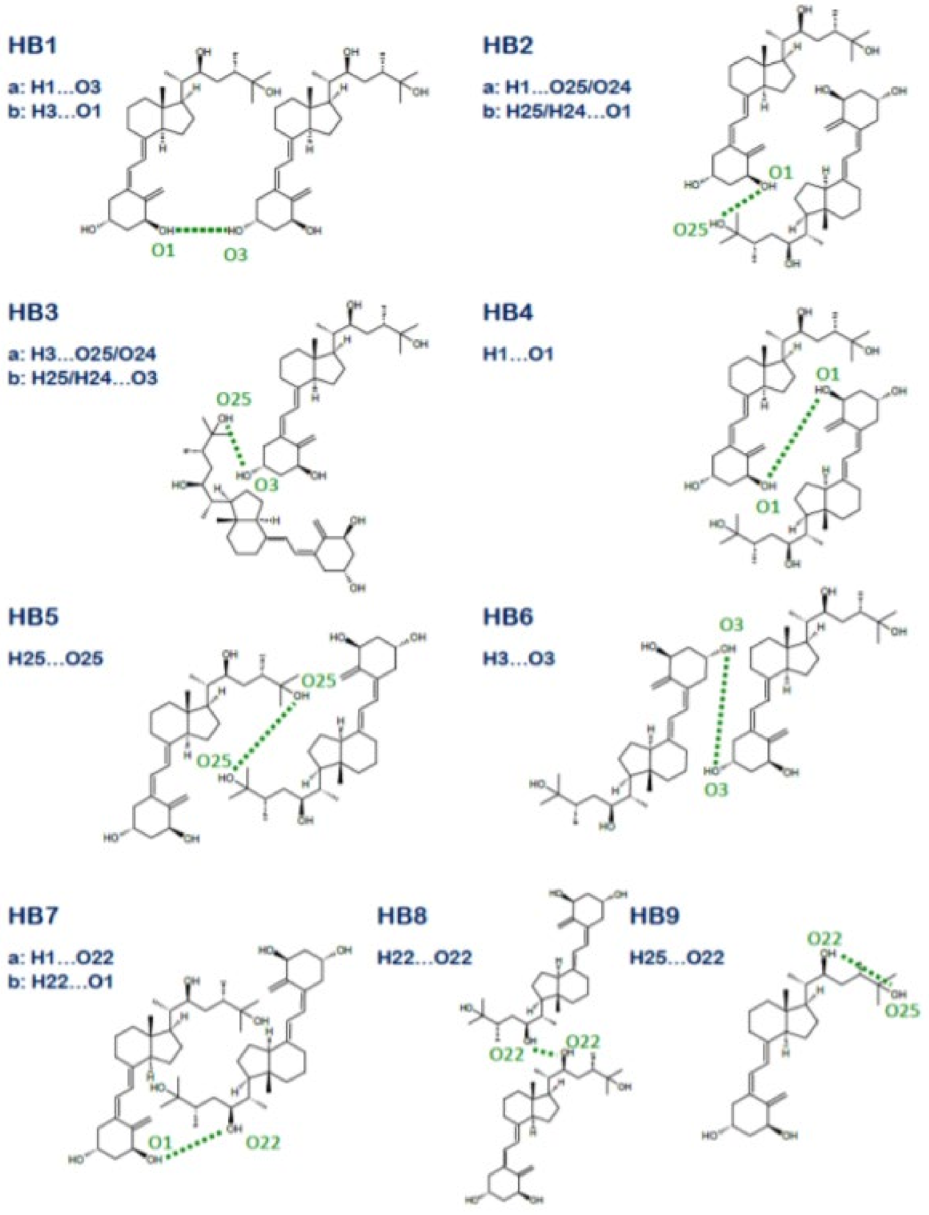

Figure S9 Selected motifs found in vitamin D analogues. Reprinted with permission from Wanat, M., Malinska, M., Kutner, A., & Wozniak, K. (2018). Effect of vitamin D conformation on interactions and packing in the crystal lattice. Crystal Growth & Design, 18(6), 3385-3396. Copyright 2018 American Chemical Society.

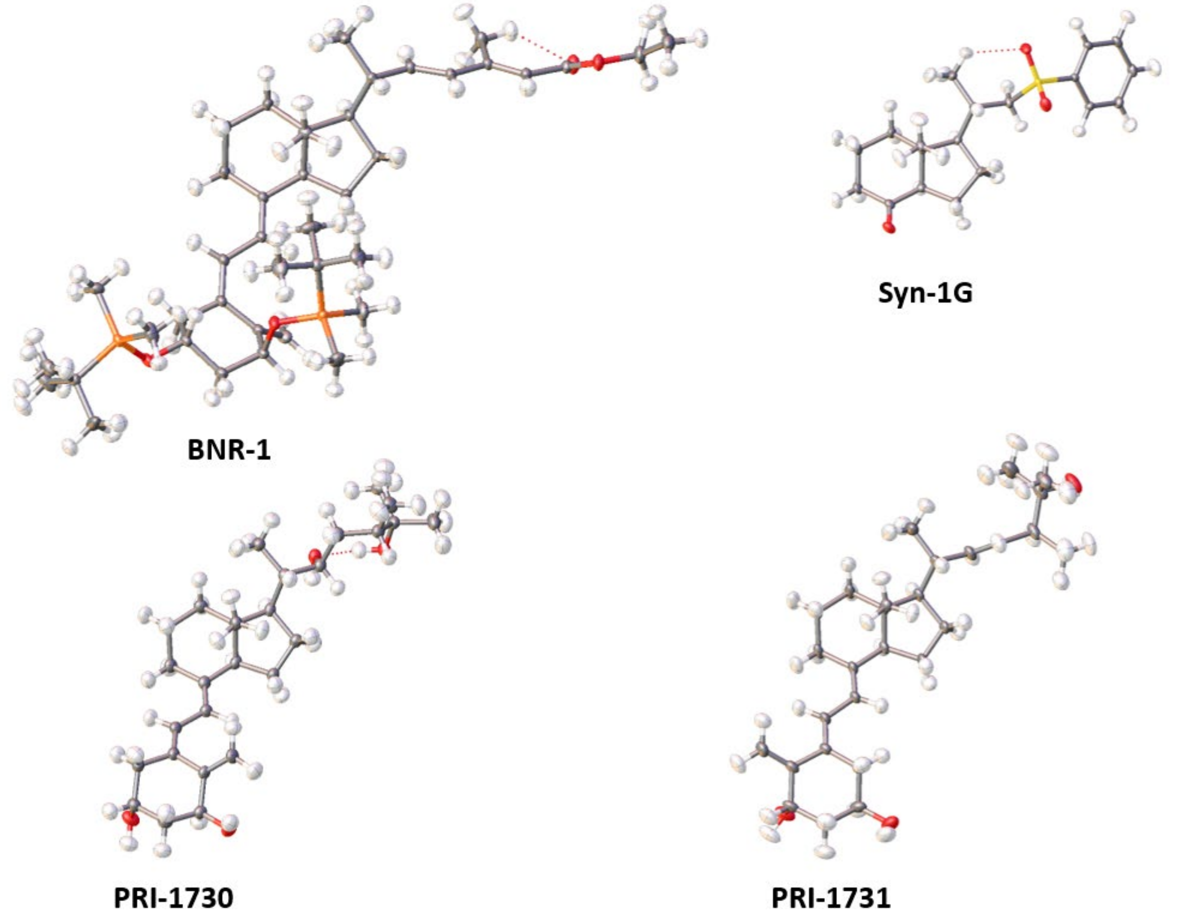

Figure S10 Displacement ellipsoids (50% probability level) of the molecular structures obtained with the MM refinements of BNR-1 and Syn-1G and TAAM refinements of PRI-1730 and PRI-1731. Numbering system is omitted for clarity and available in ms.
